# Development and validation of machine learning-based clinical decision support tool for identifying malnutrition in NICU patients

**DOI:** 10.1038/s41598-023-32570-z

**Published:** 2023-03-30

**Authors:** Nadir Yalçın, Merve Kaşıkcı, Hasan Tolga Çelik, Kutay Demirkan, Şule Yiğit, Murat Yurdakök

**Affiliations:** 1 Department of Clinical Pharmacy, Faculty of Pharmacy, Hacettepe University, 06230 Ankara, Turkey; 2 Department of Biostatistics, Faculty of Medicine, Hacettepe University, 06230 Ankara, Turkey; 3 Division of Neonatology, Department of Child Health and Diseases, Faculty of Medicine, Hacettepe University, 06230 Ankara, Turkey

**Keywords:** Paediatrics, Medical research

## Abstract

Hospitalized newborns have an increased risk of malnutrition and, especially preterm infants, often experience malnutrition-related extrauterine growth restriction (EUGR). The aim of this study was to predict the discharge weight and the presence of weight gain at discharge with machine learning (ML) algorithms. The demographic and clinical parameters were used to develop the models using fivefold cross-validation in the *software-R* with a neonatal nutritional screening tool (NNST). A total of 512 NICU patients were prospectively included in the study. Length of hospital stay (LOS), parenteral nutrition treatment (PN), postnatal age (PNA), surgery, and sodium were the most important variables in predicting the presence of weight gain at discharge with a random forest classification (AUROC:0.847). The AUROC of NNST-Plus, which was improved by adding LOS, PN, PNA, surgery, and sodium to NNST, increased by 16.5%. In addition, weight at admission, LOS, gestation-adjusted age at admission (> 40 weeks), sex, gestational age, birth weight, PNA, SGA, complications of labor and delivery, multiple birth, serum creatinine, and PN treatment were the most important variables in predicting discharge weight with an elastic net regression (R^2^ = 0.748). This is the first study on the early prediction of EUGR with promising clinical performance based on ML algorithms. It is estimated that the incidence of EUGR can be improved with the implementation of this ML-based web tool (http://www.softmed.hacettepe.edu.tr/NEO-DEER/) in clinical practice.

## Introduction

Extrauterine growth restriction (EUGR) is an important global problem of overall health in newborns. Despite scientific and technological advances in neonatal care that have reduced the morbidity and mortality rates of very preterm births, the incidence of EUGR (discharge < 10th percentile for postmenstrual age) in very low birth weight (VLBW) preterm infants (≤ 1500 g) is still more than 80%^1,2^. Even though some achieve adequate postnatal growth during the second month of life, many newborns discharge significantly smaller than expected based on intrauterine growth restriction (IUGR) rates^3^. Weight gain is one of the most important predictors for early initiation of feed and tube feeding in neonatal intensive care units (NICUs). However, there is significant diversity in weight change and EUGR among differences ranging from 50.3 to 64.5% in the past decade^4,5^. Therefore, factors that predict neonatal growth need to be understood to improve standardized growth management and outcomes among NICU patients at high risk of developing growth restriction at discharge^6^. Only recognizing the degree of EUGR by monitoring weight change and focusing on the occurring deficit should encourage clinicians to increase nutritional support to enhance recovery growth velocity with a patient-centered approach^7^. In this context, the European Society for Paediatric Gastroenterology, Hepatology, and Nutrition (ESPGHAN) recommend the implementation of specialist pediatric nutrition support teams in children’s hospitals, with their role including nutritional risk screening^8^. Due to the limited number of pediatric nutrition support teams, especially the shortage of registered dietitian nutritionists who identify and recommend treatment for malnutrition, it is important to use nutritional screening by NICU physicians and nurses to determine the nutritional requirements of newborns. Therefore, a current, practical, and specific neonatal nutrition screening tool (NNST) was created, having 89.6% sensitivity and 75.1% specificity when used on high-risk NICU patients compared to low and moderate risks^9^. However, there is a need for updated versions with machine learning (ML) that will outperform NNST, be easier and faster to use with comparable performance, and be less error-prone. Furthermore, ML in neonates not screened for nutritional assessment can increase predictive capacity, improve efficiency, reduce patient risk, and mitigate human error^10^. Very preterm newborns have a high prevalence of EUGR, with wide variations between European countries. This variability associated with birth weight when using standard postnatal growth charts complicates international benchmarking^11,12^. Therefore, since the interest in ML algorithms has been growing across various research interests, our primary goal is to predict the presence of weight gain at discharge and discharge weight with the demographical and clinical parameters prospectively obtained in patients admitted to the NICUs with ML algorithms. Then, our secondary goal is to develop the ‘NNST-Plus’ by improving the NNST with these algorithms for use in clinical practice.

## Material & methods

### Study design and population

The Institutional Review Board of Hacettepe University ethical approved this study, and written informed consent was obtained from each parent/legal guardian of the participant (decision no. 2020/11-21). Newborns (0–28 days), patients admitted to the term and preterm (< 37 weeks) NICUs as live births for at least 24 h, patients whose parents signed an informed consent form, and patients who received at least one medication or parenteral nutrition (PN) treatment during their length of hospital stay (LOS) were included in this single-center and prospective cohort study from February 2020 to June 2021.

### Data acquisition

The following information was obtained outside of routine assessment for all study populations by NICU clinicians within the first 24 h of admission: Score for Neonatal Acute Physiology with Perinatal Extension-II (SNAPPE-II) for mortality risk assessment [variables; mean blood pressure, lowest temperature, PO_2_ (mmHg)/FiO_2_ (%), lowest serum pH, multiple seizures, urine output (mL/kg.h), APGAR score, birth weight, and small for gestational age]^13^, Neonatal Therapeutic Intervention Scoring System (NTISS) for morbidity risk assessment (variables; respiratory, drug therapy, metabolic/nutrition, procedural, cardiovascular, monitoring, transfusion, and vascular access subscores)^14^, Early-onset Sepsis Risk Calculator (EOS) for sepsis risk assessment (variables; incidence of early-onset sepsis, gestational age, highest maternal antepartum temperature, duration of rupture of membrane, maternal Group B *Streptococcus* status, and type of intrapartum antibiotics)^15^, NNST for malnutrition risk assessment (variables; gestational age, birth weight, diagnosis of absent or reversed end diastolic flow on umbilical artery Doppler, diagnosis of severe intrauterine growth restriction, defined as a birth weight below the second centile on the UK-WHO growth chart, need for gastrointestinal surgery or presence of severe gastrointestinal malformation, time to regain birth weight, and maximum percentage weight loss from birth weight and minimum rate of weekly weight gain from 2 weeks of age onwards)^9^. The general, maternal, postnatal, and discharge data were collected, as seen in Table 1. Patients’ follow-up was performed daily to acquire the clinical and nutritional status via a comprehensive assessment by the multidisciplinary team.

**Table 1:** Data acquisition parameters of the study.

The information within the first 24 h upon admission	General information	Maternal information	Postnatal information	Discharge information
*Risk scores* - SNAPPE-II - NTISS - EOS - NNST	- Type of service - Sex - APGAR scores - Gestational age - Birth weight - SGA - Multiple birth - Caesarean - Diagnosis	- Age - Diagnosis - Presence of ROM - Medications - Consanguinity - Living/gravity rate	- PNA - Weight at admission - Type of MV - Duration of MV - Presence of PN - Duration of PN - Presence of Surgery - Presence of CVC - Prescription of systemic hormonal preparations	- Discharge weight - Presence of weight gain at discharge - Weight gain/day - SGA at discharge
*Laboratory tests* - Lactate - Serum creatinine - BUN - Sodium - Total bilirubin - Fasting blood glucose levels				

*SNAPPE-II* Score for neonatal acute physiology with perinatal extension-II, *NTISS* Neonatal therapeutic intervention scoring system, *EOS* Early-onset sepsis risk calculator, *NNST* Neonatal nutrition screening tool, *BUN* Blood urea nitrogen, *APGAR* Appearance, pulse, grimace, activity, and respiration, *SGA* Smaller gestational age, *ROM* Rupture of membranes, *PNA* Postnatal age, *MV* Mechanical ventilation, *PN* Parenteral nutrition, *CVC* Central venous catheter.

### Statistical analysis

Analyses were performed using the free and open-source software R (version 4.0.3, http://www.rproject.org) by an academic statistician. Continuous variables were summarised as mean (standard deviation, SD), and categorical variables were summarised as frequency (percentage, %). ML models for classification and regression were obtained using the caret^15^ package. The contribution of features to the models was revealed using the Shapley additive explanations (SHAP) approach. SHAP values and importance graphs were provided by using fastshap^16^ and shapviz^16^ packages. pROC^16^ and precrec^17^ packages were used for the calculation of the performance measures. For classification models, accuracy, sensitivity (recall), specificity, positive predictive value (precision-PPV), negative predictive value (NPV), F_1_ score, and area under ROC curve (AUROC) with 95% confidence interval were used as performance measures. Root mean square error (RMSE) and R^2^ were evaluated for regression models. In terms of reproducibility, the seed number was set at 1234. Since there were no missing values in the dataset, any missing data imputation wasn’t performed.

### Development and optimization of classification and regression models

**Figure 1:**
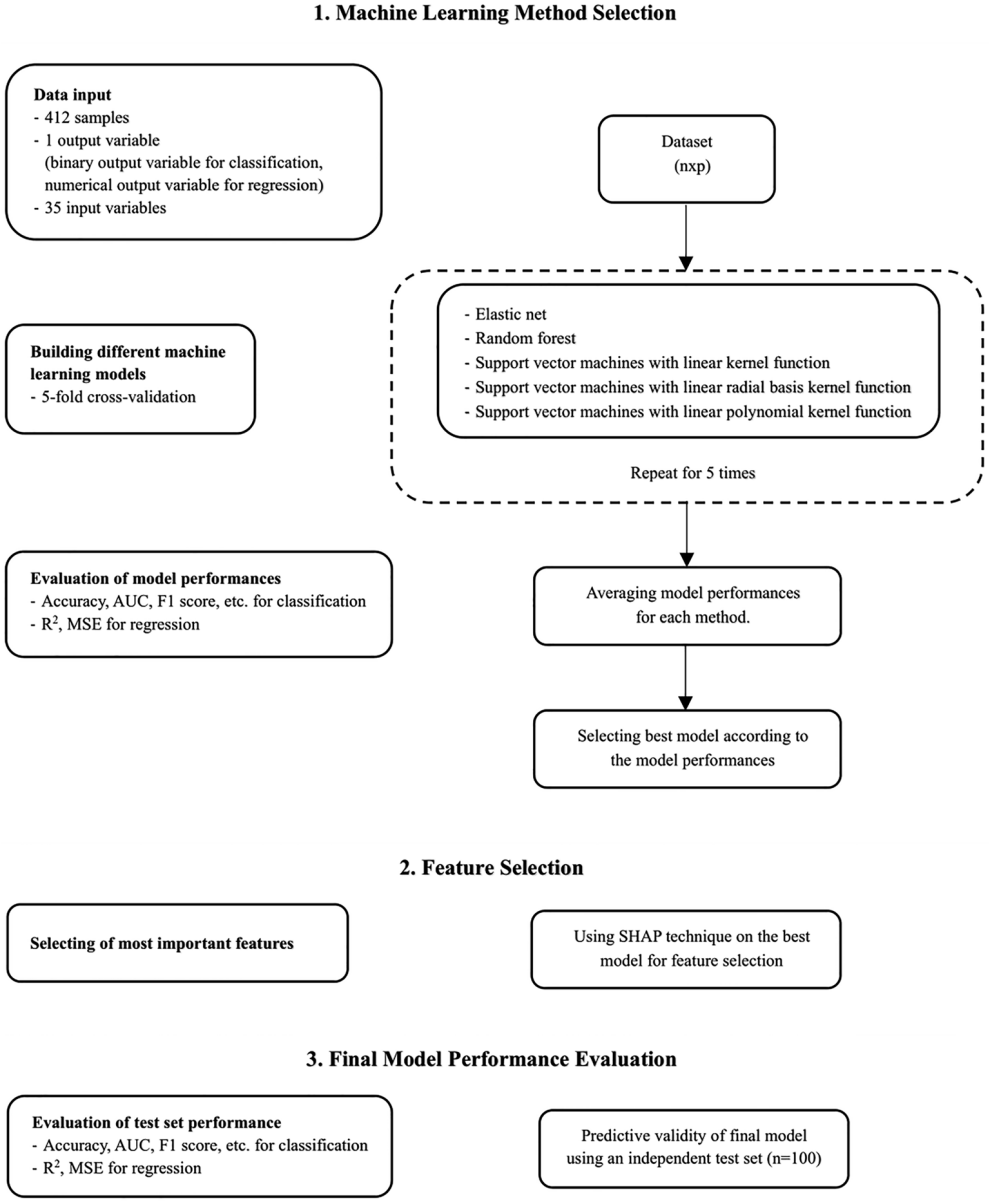
Flow chart of ML procedure.

This study aimed to develop a classification model to predict the presence of weight gain at discharge (yes or no), and a regression model to predict discharge weight (gram). The steps for classification and regression are summarized in the flow chart (Fig. 1).

The first step was selecting the most convenient ML methods for classification and regression. For this purpose, various ML methods including elastic net, random forest (RF), and support vector machines (SVM) with 3 different kernel functions (linear, radial basis, and polynomial) were used. Thirty-five variables in the dataset were entered into the model without using any variable selection. These variables were sex, APGAR scores (APGAR1, APGAR2, and APGAR3), gestational age, gestation-adjusted age at admission (< 40 weeks), SGA at admission, birth weight, multiple birth, caesarean section, diagnosis, maternal age, maternal diseases (infectious diseases, disorder of thyroid, GDM, GHT), rupture of membranes, the use of antenatal antibiotics or corticosteroids, maternal medications, consanguinity, live birth rate, PNA, weight at admission, intubation, type of MV, PN treatment, prescription of systemic hormonal preparations during hospitalization, central venous catheter, surgery, lactate, serum creatinine, BUN, sodium, total bilirubin, and LOS. Dummy variable coding was carried out for qualitative variables with more than two categories. Therefore, the number of input variables increased from 35 to 57. The quantitative features were standardized by performing the z-transform.

Following the preparation of the input variables, five different ML methods were run for classification and regression using fivefold cross-validation with five repeats. Since the fivefold cross-validation method separates the train data into train and validation sets, a separate validation set was not used when dividing the dataset. In order to prevent overfitting in ML methods, parameter optimization was made using the tuneLength argument in the caret package. The tuneLength argument provides automatic tuning of model parameters. Using this argument, alpha and lambda parameters in elastic net, number of random variables used in each tree in RF, cost parameter in SVM-linear, cost and sigma parameters in SVM-radial, cost, scale, and degree parameters in SVM-polynomial were automatically optimized.

Performance measures were calculated for each fold and each repeat. After obtaining the performance measures, average performance measures were obtained for each model. Accuracy, sensitivity, specificity, PPV, NPV, F_1_ score, and AUROC were used as performance measures in classification to compare the models. Regarding all performance measures, RF performed better than other classification methods (Supplementary Table 2). RMSE and R^2^ were calculated for regression models. Elastic net had the highest R^2^ and the lowest RMSE based on average performance measures (Supplementary Table 3).

**Figure 2:**
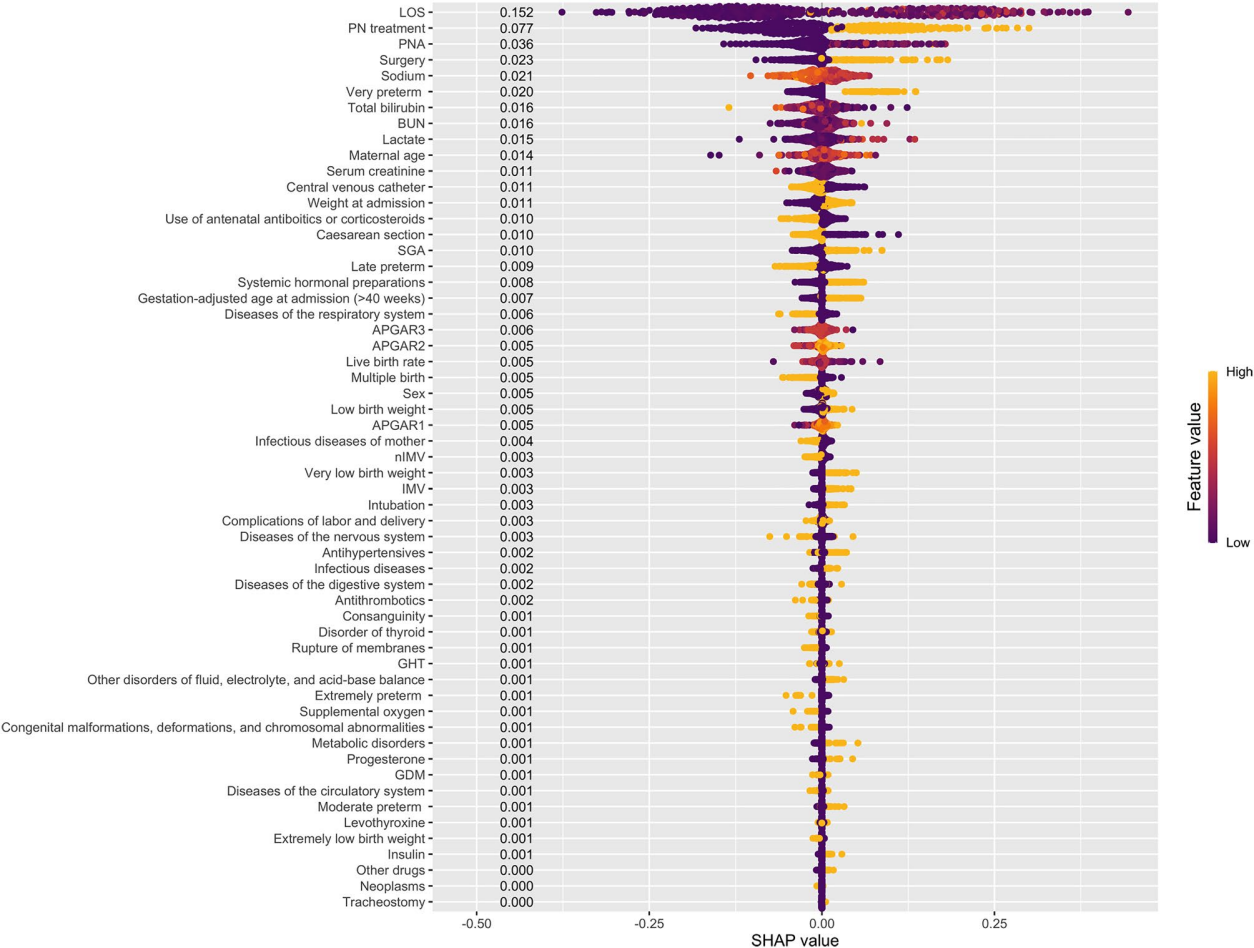
SHAP values for the presence of weight gain at discharge classification.

**Figure 3:**
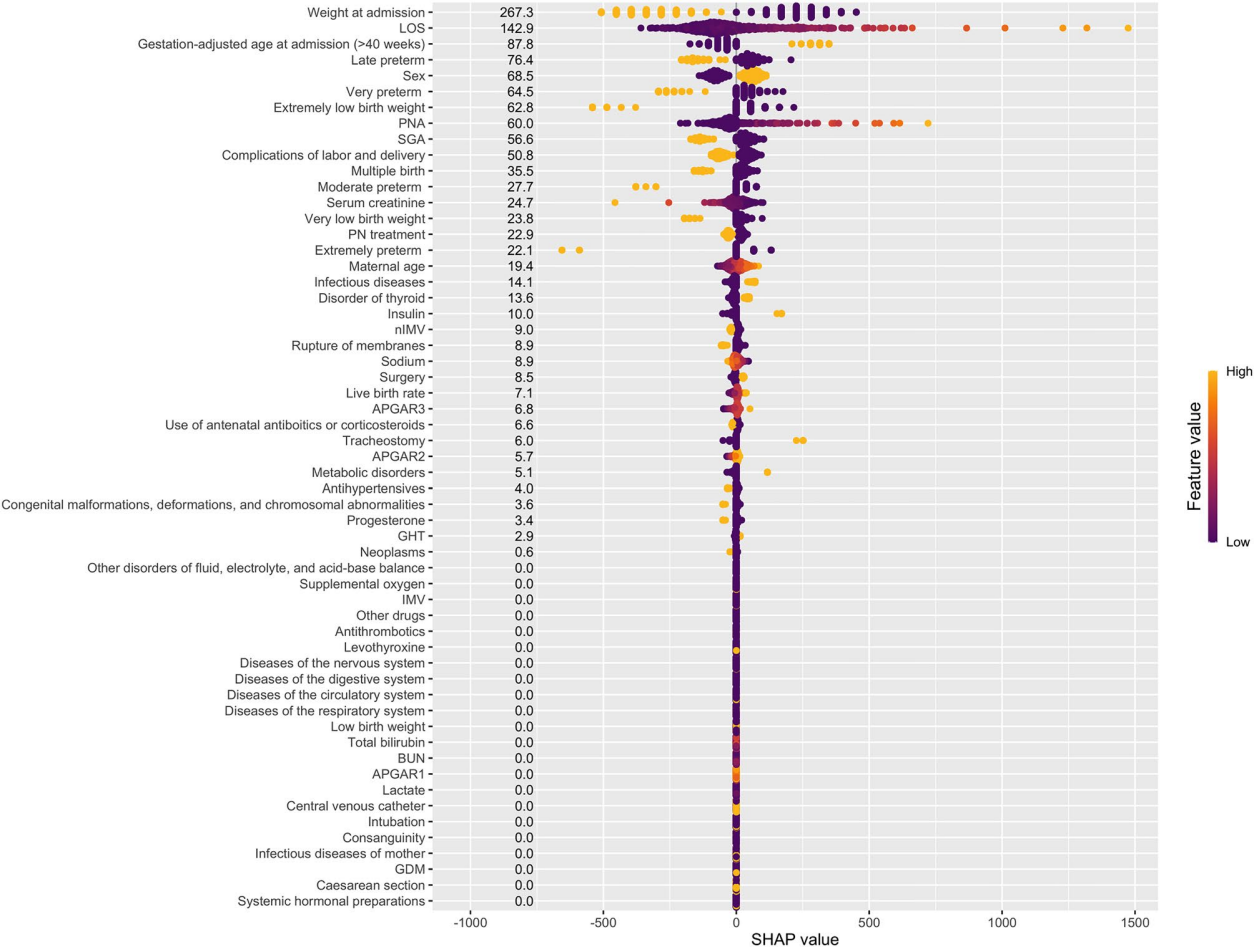
SHAP values for discharge weight prediction.

After determining ML models with the highest performance for classification and regression, the second step was selecting the most important features. The SHAP method was applied to the RF model for classification and elastic net model for regression. Since the SHAP method is a part of the explainable artificial intelligence approach, this method improves model interpretability by overcoming the black box problem in ML methods. Variable importance ranks based on the SHAP method for classification and regression are summarized in Figs. 2 and 3, respectively. For the classification model, SHAP values ranged from 0 to 0.152. We selected the top five features based on their SHAP values for the final model because including more features did not considerably impact the final model’s performance. These features were LOS, PN treatment, PNA, surgery, and sodium.

For the regression model, SHAP values ranged from 0 to 267.3. Figure 3 shows that the SHAP value of thirty-five variables was greater than zero. Since it was aimed to obtain a more compact final model in terms of duration and clinical use of the model, we considered the contribution of variables with SHAP values below 10 to the model to be very low. Therefore, we concluded that variables with SHAP values greater than 10 should be in the final model. These variables were weight at admission, LOS, gestation-adjusted age at admission (> 40 weeks), sex, gestational age (extremely preterm, late preterm, very preterm, moderate preterm), birth weight (extremely low birth weight, very low birth weight), PNA, SGA, complications of labor and delivery, multiple birth, serum creatinine, and PN treatment.

An independent test set of completely different individuals was used to assess the predictive validity of the final models. In addition, the performance of the NNST-Plus model for the presence of weight gain at discharge was compared to the NNST using the test set.

### Ethical approval

All procedures performed in studies involving human participants were in accordance with the ethical standards of the institutional and/or national research committee (The Regional Committee and Ministry of Health on Clinical Researches Ethics Boards for Turkey ref. nr 2020/11-21) and with the 1964 Helsinki declaration and its later amendments or comparable ethical standards.

### Consent to participate

Informed consent was obtained from all individual parents/legal guardians of participants included in the study.

## Results

### Baseline characteristics

During the study period, 468 newborns were admitted to the 22-bed capacity term and preterm NICUs. Fifty-six patients were excluded due to exitus (n = 21, 4.5%) and not receiving any medication or PN treatment (n = 35, 7.4%). A total of 412 patients were included in the study: There were 232 (56.3%) males, 177 (43%) preterm births, 62 (15%) gestation-adjusted age at admission (> 40 weeks), 88 (21.4%) SGA at admission, 172 (41.7%) low birth weight (< 2500 g), 167 (40.5%) low weight at admission (< 2500 g), 81 (19.7%) SGA at discharge, 145 (35.2%) low weight at discharge (< 2500 g), and 238 (57.8%) presence of weight gain at discharge in comparison to admission. PN treatment was initiated in 158 (38.3%) of the patients, and the mean duration of PN treatment was 14.36 days. In addition, the NNST risk score was determined in 92 (22.3%) patients with moderate malnutrition risk and 31 (7.5%) patients with high malnutrition risk. According to the numeric variables, the mean postnatal age (PNA) was 4.74 days, the mean LOS was 14.45 days, and the median weight gain/day was 8 g. General and postnatal information about patients and maternal information are given in Supplementary Table 1. Additionally, a total of 100 newborns were included in the study to assess predictive validity. Descriptive statistics for this data are provided in Supplementary Table 4.

### Risk assessments of hospitalization

SNAPPE-II was used to evaluate the risk of mortality (0–162 points), NTISS to assess the risk of morbidity (0–72 points), EOS to evaluate the risk of early-onset sepsis, and NNST to assess the risk of malnutrition within the first 24 h of hospitalization by using the lowest measured clinical parameters. In addition, the cut-off values for SNAPPE-II and NTISS were 37 and 23, respectively^18,19^. The majority of the patients were determined to be at low risk (49.5% and 70.1%, respectively) in terms of both EOS and NNST (Table 2).

**Table 2:** Risk scores of the study population (N = 412).

SNAPPE-II, mean (min–max)	11.83 (0–124)
SNAPPE-II, high risk (> 37 points)	43 (10.4%)
NTISS, mean (min–max)	14.16 (5–58)
NTISS, high risk (> 23 points)	38 (9.2%)
EOS, n (%)	
No risk	59 (14.3%)
Low risk (green)	204 (49.5%)
Moderate risk (yellow)	9 (2.2%)
High risk (red)	65 (15.8%)
High risk due to gestational age (< 34 weeks)	75 (18.2%)
NNST, n (%)	
Low risk	289 (70.1%)
Moderate risk	92 (22.3%)
High risk	31 (7.5%)

*SNAPPE-II* Score for neonatal acute physiology with perinatal extension-II, *NTISS* Neonatal therapeutic intervention scoring system, *EOS* Early-onset sepsis risk calculator, *NNST* Neonatal nutrition screening tool.

### Performance evaluation of the presence of weight gain at discharge model

In this and the following two subsections, the dataset with 412 observations that were used to select the best ML model and features was defined as the train set. The performance of the models was evaluated using a test set, which is summarized in Supplementary Table 4. The numerical variables in the test set were scaled with respect to the train set. The RF model had the highest performance in the prior analyses for the prediction of the presence of weight gain at discharge. Therefore, an RF classification model was performed using the top five features based on the SHAP values. The performance measures of the test set were obtained. While calculating the performance measures, the positive class was chosen as weight loss. According to the results, accuracy 0.870 (95% CI: 0.792–0.922), sensitivity 0.633 (95% CI: 0.467- 0.779), specificity 0.971 (95% CI: 0.913–0.995), PPV 0.905 (95% CI: 0.747–0.968), NPV 0.86 (95% CI: 0.806–0.902), F1 Score 0.745, and AUROC is 0.847 (95% CI: 0.746–0.950) were found.

### Comparison of the performances of NNST and NNST-plus in predicting the presence of weight gain at discharge

**Figure 4:**
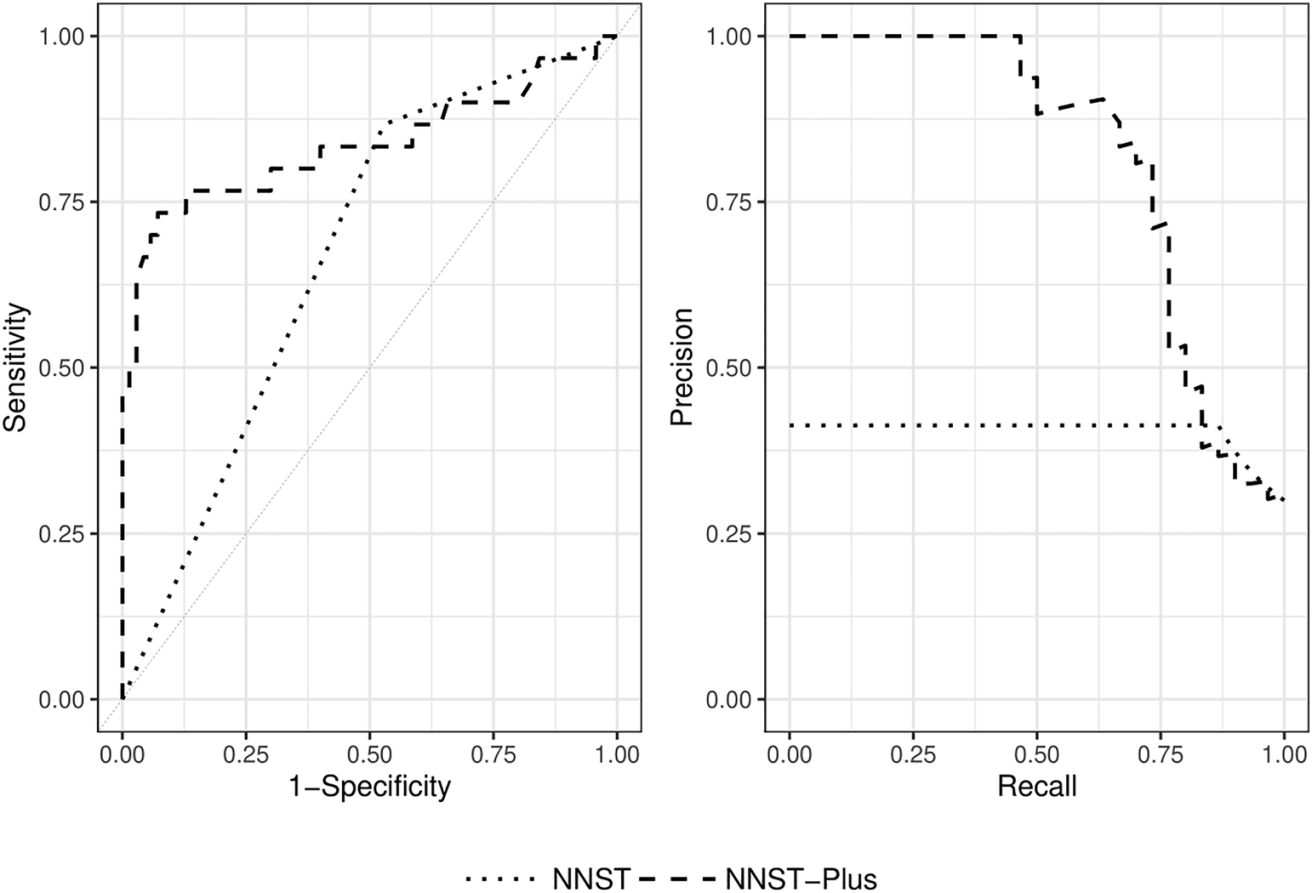
Receiver operating characteristic (ROC) curve and Precision-recall (PR) curve for weight gain at discharge classification based on NNST and NNST-plus model.

The NNST, created by Johnson et al., was insufficient to predict the presence of weight gain at discharge in clinical practice. Therefore, NNST-Plus was designed with the most important variables (LOS, PN treatment, PNA, surgery, and sodium) based on SHAP values in addition to NNST. The test set performances of these models to predict discharge weight were summarised in Table 3. NNST-Plus had higher accuracy, specificity, PPV, and AUROC compared to the NNST. These results demonstrated the superior performance of the NNST-Plus with 26% higher accuracy, 44.6% higher specificity, 48.3% higher PPV, 13.5% higher F1 score, and 16.5% higher AUROC compared to the NNST with the absolute effect (Table 3). The sensitivity of NNST was found to be quite high compared to NNST-Plus, but the specificity and PPV were quite low. According to the findings, NNST generally tended to predict 'no weight gain at discharge. When LOS, PN treatment, PNA, surgery, and sodium were added to the NNST model, the specificity and PPV significantly increased. Therefore, it was determined that the NNST-Plus model is a more useful and accurate model than the NNST in terms of prediction of the presence of weight gain at discharge. AUROC (sensitivity vs. 1-specificity curve) measured the ability to rank positive instances higher than negative ones in terms of model performance. However, in a scenario of class imbalance with a high proportion of true positive (as is the case with the prediction of ‘no weight gain at discharge’), the F1 Score and AUPRC (precision vs. recall curve) are more suitable metrics that can reflect the model’s ability to identify positive instances without regard to the negative ones. Figure 4 shows that NNST-Plus outperformed NNST in terms of AUROC and AUPRC.

**Table 3:** Comparison of classification performances of NNST and NNST-plus.

	NNST	NNST-plus	Increase/decrease absolute effect (%)	Increase/decrease relative effect (%)
Accuracy (95% CI)	0.590 (0.492–0.681)	0.850 (0.767–0.907)	+ 26.0%	+ 44.1%
Sensitivity (95% CI)	0.867 (0.720–0.953)	0.567 (0.402–0.721)	− 30.0%	− 34.6%
Specificity (95% CI)	0.471 (0.369–0.576)	0.917 (0.913–0.995)	+ 44.6%	+ 94.7%
PPV (95% CI)	0.412 (0.361–0.467)	0.895 (0.724–0.965)	+ 48.3%	+ 117.2%
NPV (95% CI)	0.892 (0.789–0.948)	0.840 (0.787–0.881)	− 5.2%	− 5.8%
F1 Score	0.559	0.694	+ 13.5%	+ 24.1%
AUC (95% CI)	0.669 (0.584–0.755)	0.834 (0.727–0.942)	+ 16.5%	+ 24.7%

### Performance evaluation of the discharge weight model

The elastic net model had the highest performance in the preliminary analyses for estimating the discharge weight. Therefore, feature selection was accomplished by calculating SHAP values based on the elastic net regression model, and the feature with the highest importance was weight at admission. In order to evaluate the elastic net model performance, RMSE and R^2^ of the test set were calculated. RMSE of the model was 428.56, and R^2^ was 0.748. This result suggests that the independent variables in the model together could explain 74.8% of the change in the discharge weight.

## Discussion

We performed a single-center, observational cohort study including 412 NICU patients with 11 common diseases. The study’s results demonstrate that the presence of weight gain was predicted at discharge with the RF model and discharge weight gain with an elastic net model. We also developed and upgraded a tool for identifying nutritional risk in NICU patients based on the NNST. Compared with the NNST, which the gestational age might only evaluate, birth weight, and presence of any digestive system diseases (congenital gastrointestinal perforation, malformation, NEC, etc.), the machine learning-based NNST-Plus had a superior performance with LOS, PN, PNA, surgery, and sodium that are the most important variables in terms of predicting the presence of weight gain at discharge, which might provide novel insights to better guide clinical practice with these web-tools. Also, because of its current, specific, evidence-based, and user-friendly nutritional risk screening, this tool might serve as a decision reference, especially for neonatologists, dietitians, or nurses, to accelerate multidisciplinary neonatal care in the NICU during hospitalization. In addition, the results showed that the most important variables in predicting discharge weight were weight at admission, LOS, and gestation-adjusted age at admission (> 40 weeks).

In the literature, estimation of growth rate, daily weight gain, and inadequate nutritional intake are of great importance for diagnosing malnutrition in preterm infants and neonates. Early aggressive nutrition and planning of PN can be predicted with this model^20^. There is evidence that changes in nutritional support can have a positive effect on growth. These include early administration of intravenous amino acids/lipids and breast milk^7^. Especially for infants at risk of EUGR at discharge, individualized breastfeeding and nutrition education can be arranged for their mothers before discharge. It is known that EUGR, which has a severe problem in the NICU, is associated with gestational age and birth weight. However, independent factors such as male sex, MV need on the first day of life, a history of NEC, and prescription of steroids during hospitalization are also included in the literature^3^. Although prediction of maternal (preeclampsia, gestational hypertension, gestational diabetes) and perinatal (IUGR, SGA, retinopathy of prematurity) outcomes with ML algorithms has been shown in the literature^21,22^, there is no study predicting EUGR with the presence of weight gain at discharge and discharge weight in NICU patients.

The strongest predictors of neonatal growth velocity are days to regain birth weight, emphasizing the importance of the first two weeks of life in growth pattern establishment^5^. In our study, even in patients hospitalized for less than 14 days, weight gain and discharge weight can be predicted with the advantage of patient heterogeneity. We recommend that this be considered when using the web tool in clinical practice. In addition, predicting weight gain and target weight at discharge is of great importance for pre-planning the feeding schedule and growth velocity calculation during hospitalization with a patient-centered approach. According to the study by Meetze, newborns with respiratory disease gain weight more slowly than other newborns^23^. A similar result was found in our study due to diseases of the respiratory system being one of the 20 most important variables in predicting the presence of weight gain at discharge (Fig. 2). On the other hand, it is known that EUGR is associated with a higher incidence of co-morbidities^24^. Furthermore, because admission to the surgical NICU following surgery is an important variable in predicting weight gain and discharge weight, an organized and focused approach to clinical nutrition (earlier breast milk fortification, evaluation of feeding tolerance, and so on) was recommended, particularly for those requiring cardiac surgery (33). Variables predictive of weight loss can facilitate the identification of high-risk neonates to prevent significant weight loss. In a study, strong predictors of weight loss were the type of feeding and number of wet diapers^25^. However, since these variables, which might be subjective and difficult to follow up, could not be used consistently in clinical practice. Therefore, we recommend using the objective variables included in our prediction models.

Futatani et al. found a strong positive correlation between inadequate breast milk intake and blood sodium levels (an accurate marker of dehydration) in the early postnatal period^26^. Similarly, we determined blood sodium level as one of the five most important variables in predicting the presence of weight gain at discharge (Fig. 3). It is known that BUN values are used during enteral and parenteral nutrition to evaluate the quantity of protein intake in preterm infants^27^. According to the study by Nagaya et al., the prediction of cognitive development at the corrected age of 36 months was observed to be significantly correlated with the corrected BUN (cBUN)^28^. In our study, BUN was also determined as one of the ten most important variables in predicting the presence of weight gain at discharge (Fig. 2). In a multicenter cohort study, females were at a slightly higher risk of postnatal weight restriction^29^. Similarly, in our study, females were at a slightly higher risk of having a lower discharge weight. In addition, the male sex was one of the five most important variables in predicting discharge weight (Fig. 3).

Machine learning methods that predict fetal growth abnormalities based on maternal information toward discharge are available in the current literature^30,31^. However, by improving the current, practical, and evidence-based NNST, the ML model that predicts whether there will be weight gain from hospitalization to discharge with high performance is not included in the current literature. In this context, the models we have developed will be beneficial in clinical practice.

The limited duration of the study and the number of patients due to prospective real data, as well as the absence of patient and health service (policy) heterogeneity due to the single-center study design, are two notable limitations.

In conclusion, this is the first study in the literature to predict the presence of weight gain at discharge using NNST and to predict discharge weight with ML algorithms. Using RF and elastic net-based machine learning, we visualized and validated a free web tool (http://www.softmed.hacettepe.edu.tr/NEO-DEER/) that can be most conveniently used to accelerate the pre-discharge determination of malnutrition in NICU patients. The superior predictive performance of NNST-Plus compared to screening based on the classical NNST may facilitate clinicians’ prioritization of patients for evaluation. We predict that increased time efficiency and higher malnutrition detection rates will improve clinicians' performance and reduce the risk of malnutrition while newborns go home.

## Supplementary Information

Supplementary Information.

## Data Availability

The datasets generated during and/or analyzed during the current study are not publicly available due to ethical restrictions but are available from the corresponding author on reasonable request.

## Code availability

The codes generated during and/or analyzed during the current study are available in the SoftMed repository, http://www.softmed.hacettepe.edu.tr/NEO-DEER/.
