# Supplementary material for: Development and validation of machine learning-based clinical decision support tool for identifying malnutrition in NICU patients
Source: Sci Rep. 2023 Mar 30;13:5227. doi: 10.1038/s41598-023-32570-z (PMC10063679; doi:10.1038/s41598-023-32570-z)
Supplement: Supplementary file 1 — Supplementary Information. [file 41598_2023_32570_MOESM1_ESM.docx]

Suppl Table 1. Baseline characteristics of the study population (n=412).

| **General information** | |
| --- | --- |
| Sex, male, n (%) | 232 (56.3%) |
| APGAR1 score, mean (SD) | 7.58 (1.96) |
| APGAR2 score, mean (SD) | 8.52 (2.05) |
| APGAR3 score, mean (SD) | 8.97 (1.96) |
| Gestational age (weeks), mean (SD) | 35.8 (3.57) |
| *Extremely preterm (<28 weeks)* | 7 (1.7%) |
| *Very preterm (28 to 32 weeks)* | 52 (12.6%) |
| *Moderate preterm (32 to 34 weeks)* | 16 (3.9%) |
| *Late preterm (34 to 37 weeks)* | 102 (24.8%) |
| *Term (>37 weeks)* | 235 (57%) |
| Gestation-adjusted age at admission (<40 weeks), n (%) | 350 (85%) |
| Gestation-adjusted age at discharge (<40 weeks), n (%) | 268 (65%) |
| SGA at admission, n (%) | 88 (21.4%) |
| SGA at discharge, n (%) | 81 (19.7%) |
| Birth weight (g), mean (SD) | 2631.1 (877.2) |
| *Extremely low birth weight (<1000 g)* | 26 (6.3%) |
| *Very low birth weight (1000 to 1500 g)* | 27 (6.6%) |
| *Low birth weight (1500 to 2500 g)* | 119 (28.9%) |
| *Normal birth weight (>2500 g)* | 240 (58.3%) |
| Multiple birth, n (%) | 53 (12.9%) |
| Caesarean section, n (%) | 337 (81.8%) |
| Diagnosis (ICD-10), n (%) |  |
| *Complications of labor and delivery* | 165 (40%) |
| *Infectious diseases* | 46 (11.2%) |
| *Diseases of the respiratory system* | 46 (11.2%) |
| *Diseases of the circulatory system* | 37 (9%) |
| *Other disorders of fluid, electrolyte, and acid-base balance* | 26 (6.3%) |
| *Diseases of the digestive system* | 24 (5.8%) |
| *Diseases of the nervous system* | 20 (4.9%) |
| *Neonatal jaundice* | 19 (4.6%) |
| *Congenital malformations, deformations and chromosomal abnormalities* | 15 (3.6%) |
| *Metabolic disorders* | 9 (2.2%) |
| *Neoplasms* | 5 (1.2%) |
| **Maternal information** | |
| Age (years), mean (SD) | 30.13 (5.8) |
| Diagnosis, n (%) |  |
| *Infectious diseases* | 106 (25.7%) |
| *Disorder of thyroid* | 67 (16.3%) |
| *Gestational diabetes mellitus (GDM)* | 53 (12.9%) |
| *Gestational hypertension (GHT)* | 49 (11.9%) |
| *Others* | 40 (9.7%) |
| Rupture of membranes, n (%) | 35 (8.5%) |
| Rupture of membranes (hours), median (min-max) (n=35) | 36.5 (5-720) |
| Medications, n (%) |  |
| *Antiboitics or corticosteroids* | 115 (27.9%) |
| *Levothyroxine* | 40 (9.7%) |
| *Antithrombotics* | 38 (9.2%) |
| *Antihypertensives* | 27 (6.6%) |

| *Insulin* | 13 (3.2%) |
| --- | --- |
| *Progesterone* | 13 (3.2%) |
| *Others* | 11 (2.7%) |
| Consanguinity, n (%) | 47 (11.4%) |
| Live birth rate (living/gravity), % | 89.3% |
| **Postnatal information** | |
| PNA (days), mean (min-max) | 4.74 (1-28) |
| Weight at admission (g), mean (SD) | 2649 (860.4) |
| *Low weight (<2500 g)* | 167 (40.5%) |
| *Normal weight (>2500 g)* | 245 (59.5%) |
| Intubation, n (%) | 71 (17.2%) |
| Type of MV, n (%) |  |
| *nIMV* | 99 (24%) |
| *IMV* | 50 (12.1%) |
| *Supplemental oxygen* | 17 (4.1%) |
| *Tracheostomy* | 5 (1.2%) |
| Duration of MV (days), mean (min-max) (n=171) | 13.76 (5-131) |
| PN treatment, n (%) | 158 (38.3%) |
| Duration of PN treatment (days), mean (min-max) (n=158) | 14.36 (7-78) |
| Prescription of systemic hormonal preparations during hospitalization, n (%) | 54 (13.1%) |
| Central venous catheter, n (%) | 267 (64.8%) |
| Surgery, n (%) | 75 (18.2%) |
| Lactate (mmol/L), mean (SD) | 2.63 (2.48) |
| Serum creatinine (mg/dL), mean (SD) | 0.68 (0.39) |
| BUN (mg/dL), mean (SD) | 10.85 (7.20) |
| Sodium (mEq/L), mean (SD) | 141.27 (5.10) |
| Total bilirubin (mg/dL), mean (SD) | 6.76 (4.33) |
| LOS (days), mean (min-max) | 14.45 (1-131) |
| Weight gain at discharge, n (%) | 238 (57.8%) |
| Discharge weight (g), mean (SD) | 2829 (750.5) |
| Weight gain/day, median (min-max) | 8 (-80 - 144) |

NICU: Neonatal Intensive Care Unit, APGAR: Appearance, Pulse, Grimace, Activity, and Respiration, SGA: Smaller Gestational Age, ICD- 10: International Statistical Classification of Diseases and Related Health Problems-10, PNA: Postnatal Age, nIMV: Noninvasive Mechanical Ventilation, IMV: Invasive Mechanical Ventilation, PN: Parenteral Nutrition, BUN: Blood Urea Nitrogen, LOS: Length of Hospital Stay

Suppl Table 2. Performance measures for classification models to predict the presence of weight gain at discharge

|  | **Elastic net** | **RF** | **SVM-Linear** | **SVM-Radial** | **SVM-Polinomial** |
| --- | --- | --- | --- | --- | --- |
| Accuracy | 0.744 | **0.788** | 0.738 | 0.754 | 0.744 |
| Sensitivity | 0.709 | **0.731** | 0.709 | 0.708 | 0.706 |
| Specificity | 0.770 | **0.831** | 0.761 | 0.791 | 0.776 |
| PPV | 0.694 | **0.762** | 0.685 | 0.713 | 0.699 |
| NPV | 0.785 | **0.809** | 0.782 | 0.788 | 0.783 |
| F1 Score | 0.698 | **0.743** | 0.694 | 0.706 | 0.698 |
| AUC | 0.740 | **0.781** | 0.735 | 0.749 | 0.741 |

Suppl Table 3. Performance measures for regression models to predict the discharge weight

|  | **Elastic net** | **RF** | **SVM-Linear** | **SVM-Radial** | **SVM-Polinomial** |
| --- | --- | --- | --- | --- | --- |
| RMSE | **393.510** | 412.288 | 418.480 | 425.470 | 426.480 |
| R^2^ | **0.729** | 0.700 | 0.698 | 0.681 | 0.680 |

Suppl Table 4. Baseline characteristics of the test set (n=100).

| **General information** | |
| --- | --- |
| Sex, male, n (%) | 50 (50%) |
| APGAR1 score, mean (SD) | 7.23 (2.15) |
| APGAR2 score, mean (SD) | 8.19 (1.69) |
| APGAR3 score, mean (SD) | 8.85 (1.37) |
| Gestational age (weeks), mean (SD) | 34.8 (3.47) |
| *Very preterm (28 to 32 weeks)* | 22 (22%) |
| *Moderate preterm (32 to 34 weeks)* | 8 (0%) |
| *Late preterm (34 to 37 weeks)* | 34 (34%) |
| *Term (>37 weeks)* | 36 (36%) |
| Gestation-adjusted age at admission (<40 weeks), n (%) | 90 (90%) |
| SGA at admission, n (%) | 30 (30%) |
| Birth weight (g), mean (SD) | 2287.7 (763.9) |
| *Extremely low birth weight (<1000 g)* | 6 (6%) |
| *Very low birth weight (1000 to 1500 g)* | 11 (11%) |
| *Low birth weight (1500 to 2500 g)* | 39 (39%) |
| *Normal birth weight (>2500 g)* | 44 (44%) |
| Multiple birth, n (%) | 14 (14%) |
| Caesarean section, n (%) | 90 (90%) |
| Diagnosis (ICD-10), n (%) |  |
| *Complications of labor and delivery* | 52 (52%) |
| *Infectious diseases* | 3 (3%) |
| *Diseases of the respiratory system* | 2 (2%) |
| *Diseases of the circulatory system* | 22 (22%) |
| *Diseases of the digestive system* | 7 (7%) |
| *Diseases of the nervous system* | 4 (4%) |
| *Metabolic disorders* | 1 (1%) |
| *Others* | 9 (9%) |
| **Maternal information** | |
| Age (years), mean (SD) | 30.3 (6) |
| Diagnosis, n (%) |  |
| *Infectious diseases* | 32 (32%) |
| *Disorder of thyroid* | 20 (20%) |
| *Gestational diabetes mellitus (GDM)* | 11 (11%) |
| *Gestational hypertension (GHT)* | 17 (17%) |
| *Others* | 12 (12%) |
| Rupture of membranes, n (%) | 7 (7%) |
| Medications, n (%) |  |
| *Antiboitics or corticosteroids* | 33 (33%) |
| *Levothyroxine* | 8 (8%) |
| *Antithrombotics* | 13 (13%) |
| *Antihypertensives* | 10 (10%) |

| *Insulin* | 1 (1%) |
| --- | --- |
| *Progesterone* | 5 (5%) |
| *Others* | 4 (4%) |
| Consanguinity, n (%) | 10 (10%) |
| Live birth rate (living/gravity), % | 84.2% |
| **Postnatal information** | |
| PNA (days), mean (min-max) | 3.6 (1-28) |
| Weight at admission (g), mean (SD) | 2689.7 (878.9) |
| *Low weight (<2500 g)* | 60 (60%) |
| *Normal weight (>2500 g)* | 40 (40%) |
| Intubation, n (%) | 24 (24%) |
| Type of MV, n (%) |  |
| *nIMV* | 27 (27%) |
| *IMV* | 31 (31%) |
| *Supplemental oxygen* | 1 (1%) |
| *Tracheostomy* | 1 (1%) |
| Duration of MV (days), mean (min-max) (n=52) | 13.85 (1-104) |
| PN treatment, n (%) | 63 (63%) |
| Duration of PN treatment (days), mean (min-max) (n=56) | 11.43 (1-85) |
| Prescription of systemic hormonal preparations during hospitalization, n (%) | 21 (21%) |
| Central venous catheter, n (%) | 80 (80%) |
| Surgery, n (%) | 36 (36%) |
| Lactate (mmol/L), mean (SD) | 2.34 (2.34) |
| Serum creatinine (mg/dL), mean (SD) | 0.79 (0.32) |
| BUN (mg/dL), mean (SD) | 13.83 (7.4) |
| Sodium (mEq/L), mean (SD) | 141.64 (4.7) |
| Total bilirubin (mg/dL), mean (SD) | 5.98 (3.5) |
| LOS (days), mean (min-max) | 27.8 (3-144) |
| Weight gain at discharge, n (%) | 70 (70%) |
| Discharge weight (g), mean (SD) | 2710.8 (799.9) |

NICU: Neonatal Intensive Care Unit, APGAR: Appearance, Pulse, Grimace, Activity, and Respiration, SGA: Smaller Gestational Age, ICD- 10: International Statistical Classification of Diseases and Related Health Problems-10, PNA: Postnatal Age, nIMV: Noninvasive Mechanical Ventilation, IMV: Invasive Mechanical Ventilation, PN: Parenteral Nutrition, BUN: Blood Urea Nitrogen, LOS: Length of Hospital Stay
